# The diagnostic performance of a novel ELISA for human CTP (Cochlin-tomoprotein) to detect perilymph leakage

**DOI:** 10.1371/journal.pone.0191498

**Published:** 2018-01-29

**Authors:** Tetsuo Ikezono, Tomohiro Matsumura, Han Matsuda, Satomi Shikaze, Shiho Saitoh, Susumu Shindo, Setsuo Hasegawa, Seung Ha Oh, Yoshiaki Hagiwara, Yasuo Ogawa, Hiroshi Ogawa, Hiroaki Sato, Tetsuya Tono, Ryuichiro Araki, Yukihide Maeda, Shin-ichi Usami, Yasuhiro Kase

**Affiliations:** 1 Department of Otorhinolaryngology, Saitama Medical University, Saitama, Japan; 2 Department of Biochemistry and Molecular Biology, Nippon Medical School, Graduate School of Medicine, Tokyo, Japan; 3 R&D and Business Development Segment, Mitsubishi Chemical Medience Corporation, Tokyo, Japan; 4 Sekino Clinical Pharmacology Clinic, Hoeikai Med.Corp., Tokyo, Japan; 5 Department of Otorhinolaryngology, Seoul National University Hospital, Seoul, Korea; 6 IBL Co., Ltd. (Immuno-Biological Laboratories Co., Ltd.), Fujioka-shi, Gunma, Japan; 7 Department of Otolaryngology, Tokyo Medical University Hachioji Medical Center, Tokyo, Japan; 8 Department of Otorhinolaryngology, Fukushima Medical University Medical Center, Aizuwakamatsu, Japan; 9 Department of Otolaryngology-Head and Neck Surgery, Iwate Medical University, Morioka, Japan; 10 Department of Otolaryngology, Faculty of Medicine, University of Miyazaki, Miyazaki, Japan; 11 Community Health Science Center, Saitama Medical University, Saitama, Japan; 12 Department of Otolaryngology-Head and Neck Surgery, Okayama University Graduate School of Medicine, Dentistry and Pharmaceutical Sciences, Okayama, Japan; 13 Department of Otorhinolaryngology, Shinshu University School of Medicine, Nagano, Japan; Kyoto Daigaku, JAPAN

## Abstract

Perilymphatic fistula is defined as an abnormal communication between the perilymph-filled space and the middle ear, or cranial spaces. The manifestations include a broad spectrum of neuro-otological symptoms such as hearing loss, vertigo/dizziness, disequilibrium, aural fullness, tinnitus, and cognitive dysfunction. By sealing the fistula, perilymphatic fistula is a surgically correctable disease. Also, appropriate recognition and treatment of perilymphatic fistula can improve a patient’s condition and hence the quality of life. However, the difficulty in making a definitive diagnosis due to the lack of an appropriate biomarker to detect perilymph leakage has caused a long-standing debate regarding its management. We have reported a clinical test for the diagnosis of perilymphatic fistula by detecting a perilymph specific protein, Cochlin-tomoprotein, as a diagnostic marker using a western blot. The aim of this study is to establish an ELISA-based human Cochlin-tomoprotein detection test and to evaluate its diagnostic accuracy in clinical subjects. The results of ELISA showed good dilution reproducibility. The mean concentration was 49.7±9.4 of 10 perilymph samples. The ROC curve in differentiating the perilymph leakage condition from the normal middle ear was significant (P < 0.001) with an area under the curve (AUC) of 0.918 (95% CI 0.824–0.100). We defined the diagnostic criteria as follows: CTP<0.4 negative; 0.4≦CTP<0.8 intermediate; 0.8≦CTP(ng/ml) positive in the clinical usage of the hCTP ELISA, and sensitivity and specificity were 86.4% and 100%, respectively. We further tested the expression specificity of the Cochlin-tomoprotein by testing blood and CSF samples. The concentration was below the detection limit (0.2 ng/ml) in 38 of the 40 blood, and 14 of the 19 CSF samples. We report the accuracy of this test for the diagnosis of perilymphatic fistula. Using ELISA, we can improve the throughput of the test. Furthermore, it is useful for a large-scale study to characterize the clinical picture and delineate the management of this medical condition.

## Introduction

Perilymphatic fistula (PLF) is defined as an abnormal communication between the fluid (perilymph)-filled space of the inner ear and the air-filled space of the middle ear and mastoid, or cranial spaces. PLF is located in the round or oval window, fractured bony labyrinth, microfissures, or anomalous foot plate, and can occur after head trauma or barotrauma, chronic inflammation, or in otic capsule dehiscence [1] [2] [3] [4].

If there is a leakage of perilymph from fistulization, the symptoms may fluctuate and worsen. Although these potential pathways exist between the perilymphatic space and the middle ear, the actual leaking of fluid can be difficult or impossible to prove. The difficulty of making a definitive diagnosis of PLF due to the lack of an appropriate biomarker to detect perilymph leakage has caused a long-standing debate regarding its prevalence, natural history, management, and even its very existence [5], [6], [7], [8], [9].

The primary manifestations of perilymph fistulization are sudden or progressively, fluctuating sensorineural hearing loss and vertigo/dizziness. Other symptoms that may be present include a broad spectrum of neuro-otological symptoms such as tinnitus, disequilibrium, and aural fullness, and cognitive dysfunction [1], [2], [10], [11]. PLF is surgically correctable by sealing the fistula, and appropriate recognition and treatment of PLF can improve these symptoms, and in turn, improve the quality of life of afflicted patients. Therefore, PLF is an especially important and treatable disease for otologists.

Based on the proteomic analysis, we have identified an isoform of Cochlin, (Cochlin-tomoprotein (CTP) [12]), as a perilymph-specific protein that is not expressed in the blood, CSF or saliva [13]. The detection of CTP in the middle ear indicates the presence of a perilymphatic fistula and perilymph leakage. We have performed a western blot (WB) based test for CTP detection [13] [12] [14]. In the case of post-stapes surgery hearing loss due to dislocation of the piston, the CTP detection test could be used to identify perilymph leakage before exploratory tympanotomy [15], while in the case of direct middle ear trauma, the CTP test was useful in deciding surgical treatment [16]. Although it is well known, there are some drawbacks in the WB detection system, such as it requires a relatively large volume sample, handles small numbers of samples at one time, and its ability to identify “the positive band” depends on subjective judgment.

The aim of this study is to establish an ELISA-based human CTP detection test (hereafter called hCTP ELISA) and to evaluate the accuracy of CTP detection from the middle ear in clinical subjects and to diagnose perilymph leakage. Moreover, using this hCTP ELISA, we hope to further prove the expression specificity of CTP by testing blood samples. Our report delineates the accuracy and the usefulness of the CTP detection test for the diagnosis of PLF. An ELISA-based test can improve the throughput of the test and it is useful for a large-scale study to characterize the clinical picture and delineate the management of this medical condition.

## Materials and methods

The study was approved by the Ethics Committee of Saitama Medical University, as well as the relevant bodies of the participating institutions. The approval numbers of each institution are as follows: Saitama Medical University 13086; Seoul National University Hospital, 0904-039-27; Fukushima Medical University, 915; Iwate Medical University, H2481; Tokyo Medical University, 1644; University of Miyazaki (Faculty of Medicine), 201399; and Sekino Clinical Pharmacology Clinic (Houeikai Med. Corp), 12000088. All subjects gave their written informed consent to participate by recommendations in the Declaration of Helsinki.

### Production of hCTP ELISA, evaluation of the test and comparison with WB

We have developed hCTP ELISA for the automated, objective measurement of large-scale samples. Also, we have compared the ELISA measurement with previously performed WB CTP assay conducted in our laboratory.

### Production of standard CTP protein for WB and ELISA

The recombinant human rhCTP (E.Coli) was used as a standard protein. The exact N and C-terminal sequence of CTP is not yet known. However, in a putative CTP sequence predicted from our previous study [14], the 101 to 403 position of the cDNA corresponding to the amino acid residues 32–132 was amplified by PCR from a human expressed sequence tag clone, IMAGE ID 27789 (Kurabo, Japan). According to the manufacturer’s protocol, rhCTP was produced in a final concentration of 0.17 mg/ml using pCR/T7/TOPO/TA Expression Kits (Invitrogen).

### Peptide synthesis, immunization, and antibody purification

Using the amino acid sequence of the human Cochlin protein deduced from the corresponding gene sequence, we designed antigenic peptides with the Epitope Advisor program (Fujitsu Kyushu System Engineering) to generate CTP-specific antibodies. Peptide #1, which was used to generate anti-hCTP-A, was a 16-mer (CFTRGLDIRKEKADVL) corresponding to residues 34–49. Peptide #2, which was used to generate anti-hCTP-B, was an 18-mer (RVYSLPGRENYSSVDANG) corresponding to residues 91–111. Peptide #3, which was used to generate anti-hCTP-C, was a 15-mer (SASFTVTKGKSSTQE) corresponding to residues 118–132. Rabbits were immunized by an conventional method, and the serum was purified by a protein A column, followed by peptide affinity chromatography. The specificity of the antibodies for the corresponding antigenic peptides was confirmed by dot blot analysis and a peptide absorption test [12].

### Production of hCTP ELISA

An Immuno Module Plate (Nalge Nunc, Rochester, NY, USA) was coated with a mixture of anti-hCTP-A and anti-hCTP-B (in 0.1 mol /L carbonate buffer, pH 9.5) and incubated at 4°C overnight, then blocked with 1% bovine serum albumin in PBS. The samples and standard rhCTP proteins were diluted to 10-fold with a dilution buffer (0.05% Tween 20 in PBS) and 100ul samples were added to each well, followed by incubation of the well at 37°C for 1 h. After nine washes with a washing buffer, 100 μL of horseradish peroxidase-labeled anti-hCTP-C antibody was added to each well, followed by incubation for 30 min at 4°C. After nine washes with the washing buffer, 100 μL of tetramethyl benzidine buffer was added as a substrate to each well, followed by incubation for 30 min at room temperature in the dark. Color development was stopped by the addition of 100 μL of stop solution (1 N H_2_SO_4_). The optical density of each sample at 450 nm was then measured. Samples were measured in duplicate, and serially diluted rhCTP (E.Coli) were used to make a standard curve. The concentration of CTP was calculated from the standard curve by the linear regression method. The range for the measurement was between 0.2 to 1.56 ng/ml and these are defined as the lower and upper detection limits. If the measurement was below 0.2, the result was described as 0.2. The intra- and inter-day precision, inter-person reproducibility was evaluated and confirmed to be acceptable.

### Detecting CTP by western blot analysis

WB analysis for CTP detection was performed as previously described [12, 13]. In brief, the anti-CTP antibody was prepared using 14-mer peptide #4 (LSRWSASFTVTKGK) corresponding to residues 114–127. The primary antibody (anti-CTP antibody) diluted at 1:2000 and the secondary antibody (HRP-labeled goat anti-rabbit IgG) diluted at 1:10000 were used. The detection limit of the serially diluted rhCTP was between 0.27 and 0.13 ng/well. These two amounts of rhCTP were set as the high- and low-spiked standards, respectively, and the amounts were electrophoresed each time when we tested the samples.

To evaluate the dilution reproducibility of ELISA and to measure the CTP concentration of perilymph, we collected leaked perilymph itself from cochleostomy during cochlear implantation surgery from 3 cases. Perilymph was serially diluted with saline by two-fold (8 samples) and each sample contained the following amounts of perilymph (ul): 2.0, 1.0, 0.50, 0.25, 0.125, 0.062, 0.031, and 0.015. In addition, the same perilymph was tested by WB. 2ul of perilymph was mixed with 98ul dilution buffer and it was serially diluted by two-fold (7 samples) and each sample contained the following amounts of perilymph: 1.833, 0.917, 0.458, 0.229, 0.115, 0.057, and 0.029ul/lane. The perilymph volume required to show comparable intensity with the high-spiked standard was measured. In addition, perilymph was collected from 7 cases to measure the CTP concentration by ELISA.

### Diagnostic accuracy of hCTP ELISA in detecting surgical perilymph leakage

To detect perilymph leakage in the middle ear, we used samples collected by lavaging the middle ear cavity with 0.3ml of saline and recovering the fluid, defined as middle ear lavage (MEL) [13]. To evaluate the diagnostic accuracy of hCTP ELISA in a clinical setting, MEL were collected during surgery from the 4 different middle ear conditions listed below. Samples were centrifuged at 1250g for 1min and the supernatants were frozen and stored at -80°C.

We have included 22 cases which had undergone cochlear implant surgery by a round window approach, and where a surgical fistula was made in the round window membrane for the insertion of electrodes. We excluded cases that had "cochleostomy" where a surgical fistula was made through the lateral wall of the cochlea, there was a revision cochlear implantation or an ossified cochlea, or there was a condition associated with an infection of the middle ear. We did not observe a profuse leakage of perilymph and/or CSF (i.e., a ‘gusher or oozer’) in these cases. MEL was collected in each of the 3 conditions during cochlear implantation surgery, with a set of samples (A, B, and C) collected from each of the cases:

*Sample A*: MEL was collected as soon as entering the middle ear without performing any manipulation to the cochlea and before opening the round window membrane.

*Sample B*: To create good accessibility to the round window membrane for electrode insertion, it is routine procedure to drill the round window bony overhang without breaking the membrane. With sample B, MEL was collected just after drilling the round window bony overhang.

*Sample C*: MEL was collected after electrode insertion and sealing the round window with connective tissue.

In addition to the above, we have included an additional 24 cases that had undergone exploratory tympanotomy for conductive hearing loss (middle ear ossicle anomaly or otosclerosis).

*Sample D*: MEL was collected as soon as entering the middle ear without performing any manipulation to the ossicles.

The Kruskal-Wallis test and the Dunn's multiple comparison test were used to analyze the differences in samples A, B, C and D. MEL sampling from normal subjects were not performed for ethical reasons. We considered samples A and D as controls, whereas C was considered to be the perilymph leakage condition after electrode insertion. The receiver operating characteristic (ROC) curve was constructed from CTP in differentiating the perilymph leakage condition (C) from the controls (A and D) to determine the area under the curve (AUC) as a measure of predictive accuracy and Youden’s index was used to identify the optimal cutoff value for the biomarker [17] [18]. A statistician using SPSS and Medcalc version 16 performed all the statistical analyses.

### CTP detection in blood, CSF samples

Previous WB analysis showed there was no CTP expression in the serum, CSF, saliva samples [13]. In the clinical setting, blood contamination in the MEL is unavoidable. Therefore, we have tested both serum and plasma from 40 healthy volunteers (age 25 to 64) to clarify that these samples contained CTP. Since CSF can be the source for perilymph and it can also leak out from the inner ear in a pathological condition known as “a gusher” [19], we also measured 19 CSF samples taken from suspected meningitis patients.

## Results

### Production of hCTP ELISA, evaluation of the test, and comparison with WB

**Fig 1:**
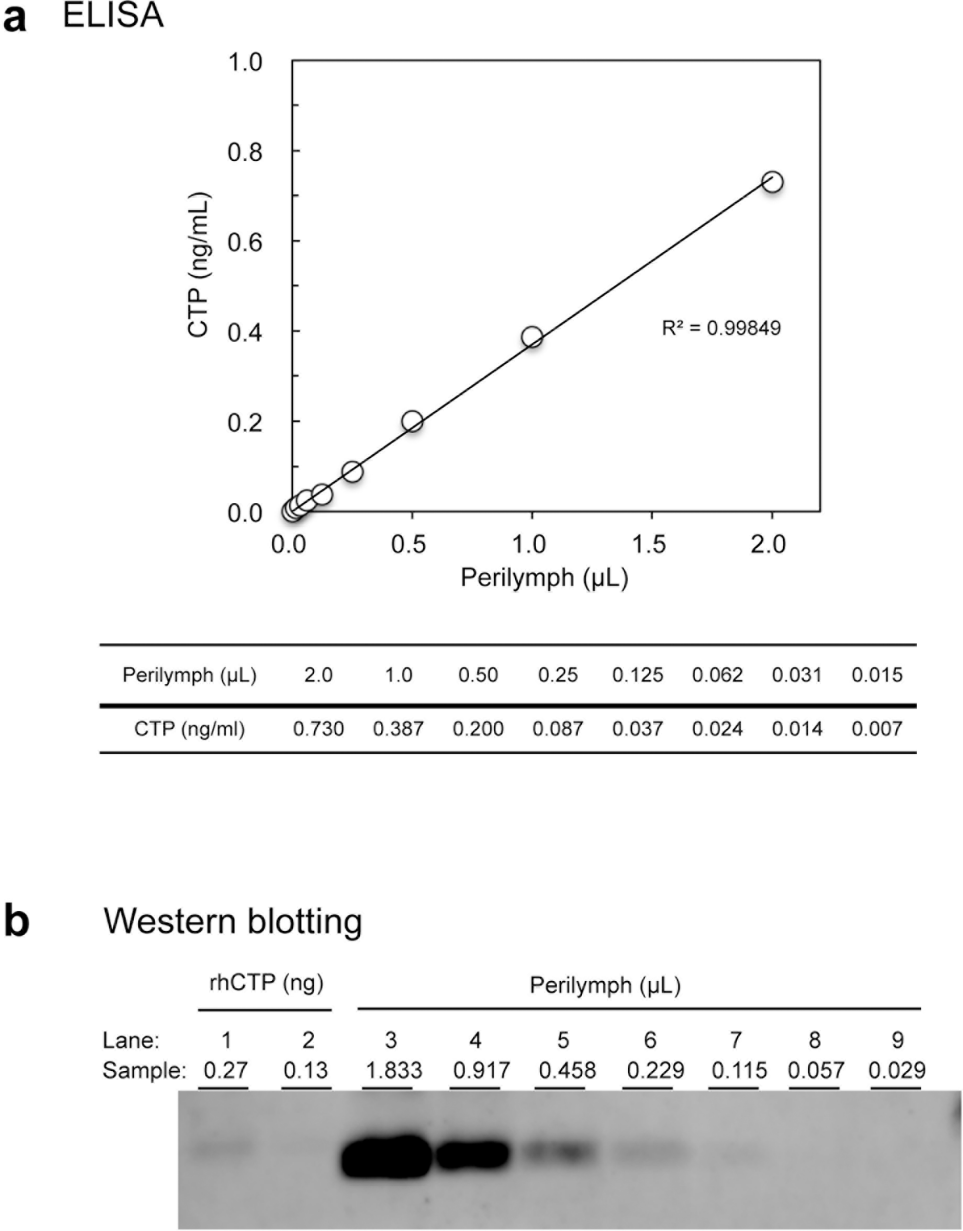
The dilution reproducibility of CTP in ELISA measurement, the CTP concentration of perilymph, and comparison with WB. We have collected leaked perilymph from the round window during cochlear implantation surgery from 3 cases, and the figure shows the representative results. Perilymph was serially diluted with saline by two-fold (8 samples) and each sample contained the following amounts of perilymph (ul) per well in ELISA: 2.0, 1.0, 0.50, 0.25, 0.125, 0.062, 0.031, and 0.015. The same perilymph was tested by WB. 2ul of perilymph was mixed with 98ul dilution buffer and it was serially diluted by two-fold (7 samples) and each sample contained the following amounts of perilymph: 1.833, 0.917, 0.458, 0.229, 0.115, 0.057, and 0.029ul/lane. The perilymph volume required to show comparable intensity with the high-spiked standard was determined. (A) The results of ELISA showed good dilution reproducibility (R^2^ = 0.99849), and the concentration of the perilymph was calculated to be 37.1 ng/ml. (B) The same perilymph was subjected to WB analysis and the perilymph volume required to show comparable intensity with a high-spiked standard of 0.229ul (lane 6) was determined.

We measured serially diluted perilymph from 3 subjects both by the ELISA and WB. The representative data is shown in Fig 1. The results of ELISA showed good dilution reproducibility (R^2^ = 0.99849), and the concentration of the perilymph was calculated to be 37.1ng/ml. The same perilymph was subjected to WB analysis and the perilymph volume required to show comparable intensity with a high-spiked standard of 0.229ul was determined (Fig 1). As for the other two perilymph samples, the concentrations were 64.4 and 57.6 ng/ml, and 0.115μL of perilymph was required to show a comparable intensity with a high-spiked standard in both of the perilymph samples. We have measured an additional 7 perilymph samples and the mean and standard deviation of 10 samples were 49.7±9.4ng/ml.

### The diagnostic accuracy of hCTP ELISA in detecting surgical perilymph leakage

**Fig 2:**
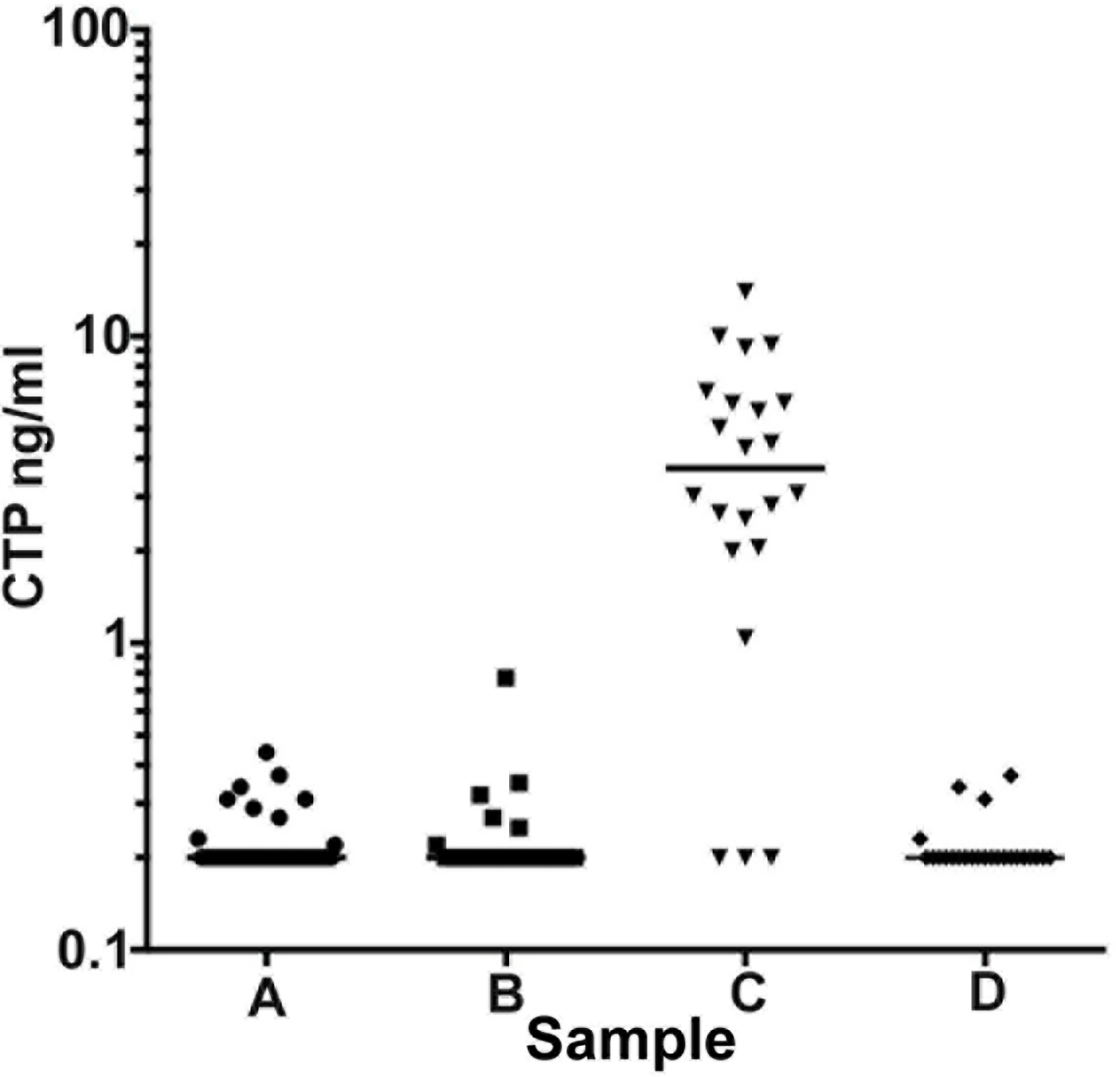
Scatter gram of CTP concentration of each group of samples. Samples A, B and C were collected from 22 cases who had undergone cochlear implant surgery. *Sample A*: MEL was collected as soon as entering the middle ear without performing any manipulation to the cochlea and before opening the round window membrane. *Sample B*: MEL was collected just after drilling the round window bony overhang. *Sample C*: MEL was collected after electrode insertion and sealing the round window with connective tissue. In addition to the above, we have included an additional 24 cases that had undergone exploratory tympanotomy for conductive hearing loss. *Sample D*: MEL was collected as soon as entering the middle ear without performing any manipulation to the ossicles. The CTP concentration of each sample was plotted and the median concentration was represented using a bar. There were statistically significant differences between sample A and C (P<0.001), B and C (P<0.001), C and D (P<0.001). No statistical differences were detected between sample A and B (P>0.99), A and D (P>0.99), B and D (P>0.99) (based on the Kruskal-Wallis test and Dunn’s multiple comparison test). In sample A, B and D, no samples contained a CTP concentration of more than 0.8. Whereas in sample C, 19 samples (86.4%) were more than 0.8. (data shown in Table 1A).

The CTP concentration of each group was blotted in Fig 2. There were statistically significant differences between sample A and C (P<0.001), B and C (P<0.001), and C and D (P<0.001). No statistical differences were detected between sample A and B (P>0.99), A and D (P>0.99), or B and D (P>0.99) (based on the Kruskal-Wallis test and Dunn’s multiple comparison test).

**Table 1:** Summaries of the number of negative, intermediate, and positive CTP cases in each group of middle ear lavage samples.

**Table 1 (a)**					
	**CTP test**				**Median**
**Sample**	**Negative**	**Intermediate**	**Positive**	**Total**	**CTP conc(ng/ml)**
**A**	**21**	**1**	**0**	**22**	**0.2**
**B**	**20**	**2**	**0**	**22**	**0.2**
**C**	**3**	**0**	**19**	**22**	**3.72**
**D**	**24**	**0**	**0**	**24**	**0.2**
**Table (b)**					
	**CTP test**				
**Sample**	**CTP negative**	**CTP positive**			
**control (A, D)**	**45**	**0**			
**perilymph leakage (C)**	**3**	**19**			

(a)The characteristics of CTP concentration for each group of samples.

The number of samples classified as negative, intermediate and positive are listed. In samples A, B and D, the majority of the samples were 0.2ng/ml (which is the detection limit of this hCTP ELISA), 95.5% (21/22), 90.9% (20/22) and 100% (24/24), respectively. None of the samples had a CTP concentration of more than 0.8. Whereas only 3 of the 22 (13.6%) samples in sample C were 0.2, and 19 of the samples (86.4%) were more than 0.8. The median of the CTP concentration was calculated in each group of samples: sample A was 0.2, B was 0.2, C was 3.72, and D was 0.2 ng/ml.

(b)x2 table of the hCTP ELISA detection test. Excluding the intermediate results, the sensitively and specificity of the test to detect perilymph leakage was 86.4% and 100%, respectively.

**Fig 3:**
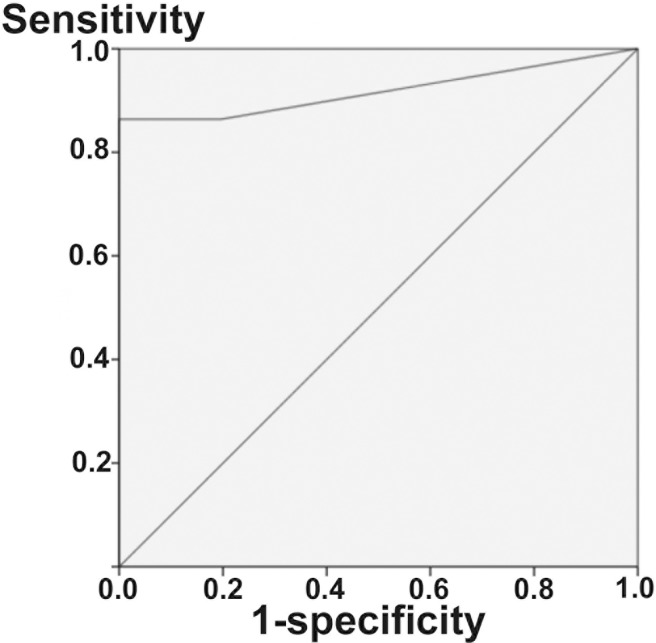
Receiver-operating characteristic (ROC) plots. A receiver operating characteristic (ROC) curve constructed from CTP in differentiating perilymph leakage conditions (Sample C) from normal middle ear conditions (Samples A and D) was significant (P < 0.001) with an area under the curve (AUC) of 0.918 (Fig 3). We then identified the optimal cutoff values using a ROC analysis with Youden’s index for CTP, and the analysis indicated no.1 = 0.740 ng/ml, (Index 0.864), and no.2 = 0.405 ng/ml, (Index 0.842). We defined the diagnostic criteria as follows: CTP<0.4 negative; 0.4≦CTP<0.8 intermediate; and 0.8≦CTP positive.

The ROC curve constructed from the CTP in differentiating the perilymph leakage condition (C) from the controls (A and D) was significant (P < 0.001) with an area under the curve (AUC) of 0.918 (95% CI 0.824–0.100, Fig 3). We then identified the optimal cutoff values using an ROC analysis with Youden’s index for CTP, and this analysis indicated that the cutoff value of 0.740 ng/ml presented a maximum Youden’s index value (0.864). The cutoff value of 0.405 ng/ml showed the second largest Youden’s index value (0.842). Based on the above results, in the clinical usage of the hCTP ELISA, we defined the diagnostic criteria as follows: CTP<0.4 negative; 0.4≦CTP<0.8 intermediate; and 0.8≦CTP(ng/ml) positive.

We determined the intermediate value as significant since MEL and CTP are both newly developed sampling methods and biomarkers, and we are still evaluating the factors that might make a false-negative or positive reactions in hCTP ELISA. Therefore, we believe that it is safe to have 3 criteria for diagnosis: negative, intermediate and positive. The number of samples analyzed based on this cutoff criterion is summarized in Table 1A. In samples A, B and D, the majority of the samples were 0.2ng/ml (which is the detection limit of this hCTP ELISA), 95.5% (21/22), 90.9% (20/22) and 100% (24/24), respectively. None of the samples had a CTP concentration of more than 0.8. Whereas only 3 of the 22 (13.6%) samples in sample C were 0.2, and 19 of the samples (86.4%) were more than 0.8. The median of the CTP concentration was calculated in each group of samples: sample A was 0.2, sample B was 0.2, sample C was 3.72, and sample D was 0.2ng/ml. By excluding the intermediate results, the sensitively and specificity of the test to detect perilymph leakage was 86.4% and 100%, respectively (Table 1B).

### CTP detection in blood and CSF samples

The CTP concentration of both plasma and serum was below the detection limit (0.2 ng/ml) in 38 of 40 of the volunteers. Two volunteers showed measurable concentrations: one was 0.45 and 0.34, and the other showed 0.31 and 0.28(ng/ml), respectively. The mean and standard deviation of the concentration was 0.21±0.04, and 0.20±0.02ng/ml, respectively. The CTP concentration of CSF was below the detection limit (0.2 ng/ml) in 14 of 19 samples. Four samples showed measurable concentrations: 0.20, 0.22, 0.23, 0.26 and 0.26 (ng/ml) and the mean and standard deviation of the concentration were 0.22±0.04 ng/ml.

## Discussion

The clinical entity of PLF was initially proposed more than a century ago, yet it has remained a topic of controversy for more than 50 years [5]. It is noteworthy that, unlike other causes of sensorineural hearing loss and dizziness, PLF is surgically correctable by sealing the fistula. We initially developed a western blotting-based CTP detection test for PLF diagnosis, and showed the clinical usefulness of this biomarker for decision making in surgical treatment [15] [16], as well as characterizing the biochemical property of the fluid [19] leaking out from cochleostomy (a gusher). In the present study, we aimed at improving the throughput and sensitivity of this test by using an ELISA-based CTP detection kit to delineate the characteristics of PLF cases. The purpose of this experiment was to establish hCTP ELISA and evaluate the accuracy of the test when measuring human perilymph. This test has accurate dilution reproducibility (Fig 1), and intra- and inter-day precision, inter-person reproducibility. We also have shown that the newly developed ELISA is comparable with the conventional WB analysis we have been utilizing.

There is no gold standard to detect perilymph leakage at present. Therefore, we have collected MEL to compare 2 middle ear conditions. One is a surgically created PLF where perilymph leaks out from the round window incision either spontaneously or after electrode insertion (sample C). The other is a presumably “normal middle ear” condition in respect to the lack of perilymph leakage, such as before cochlear implant electrode insertion (sample A), conductive hearing loss due to anomalous ossicles, or otosclerosis (sample D). In this experimental setting, the ROC curve constructed from CTP in differentiating perilymph leakage conditions from a control was significant with an AUC of 0.918, which indicates the very high diagnostic accuracy of this test. Using the criterion defined by the ROC analysis with Youden’s index for CTP, and optimizing the sensitivity and specificity, we have defined the cutoff criteria as: CTP<0.4 negative; 0.4≦CTP<0.8 intermediate; and 0.8≦CTP(ng/ml) positive. This cut-off value was used as diagnostic criteria when we performed a nationwide multicenter study of PLF in Japan [20]. By excluding the intermediate results, the sensitivity and specificity of the test to detect perilymph leakage was 86.4% and 100%, respectively. In samples A, B and D, the majority of the samples were 0.2ng/ml (which is the detection limit of the hCTP ELISA). The reason why we found 3 out of 22 (13.6%) samples in sample C was because 0.2 were unknown. However, in some cases of cochlear implantation surgery, we found perilymph does not come out from the opening of the round window due to the relative-lower pressure of perilymphatic space.

To capture a small volume of perilymph leakage from the middle ear cavity, various methods have been reported such as using gelfoam or liquids, detecting previously reported biomarkers such as beta 2 transferrin [21], or beta-trace protein [22]. In this report, we have used a standardized sampling method that is easy to perform, and involves lavaging the middle ear cavity with 0.3ml saline, (i.e., MEL) [13]. Since the hCTP ELISA is highly sensitive and specific in detecting CTP, we could use a relatively large volume of saline (i.e., 0.3ml) to lavage the middle ear, which enabled us to perform the test easily in a outpatient clinic setting after conventional myringotomy [16] [20].

Some limitations of the hCTP ELISA test warrant discussion. MEL may contain various kinds of substances and we have not fully elucidated yet what kind of components might cause a false-negative or positive reaction in the present hCTP ELISA. Another factor we need to consider is the CTP in the blood. Even though it was a small amount (0.28~0.45ng/ml) and detected only from 2 out of 40 volunteers, CTP was detectable in human serum/plasma. The possible existence of CTP in the blood of these concentrations may have a minimum effect on the positive/negative judgment of the test because the blood contamination is diluted with a much larger volume of saline when taking MEL. In CSF, the CTP concentration was below the detection limit in 14 of 19 samples and the mean was 0.22±0.04 ng/ml. This result is consistent with our previous western blot study which showed the exclusive expression of CTP in the perilymph, and blood, CSF and perilymph are discrete in nature [19].

When introducing this novel hCTP ELISA test to clinics we need to perform a cost-benefit analysis about this issue. Our previous nationwide study revealed that the incidence of CTP positive cases in suspected PLF cases linked to category 2, 3 and 4 was not negligible (approximately 20%). This test will be beneficial to a daily clinic, helping contribute to a correct and prompt diagnosis, and curing the patients using a disease-specific treatment.

In this report, we presented a novel hCTP ELISA assay as an objective method to identify CTP with high sensitivity and specificity. Coupled with this simple MEL sampling method, an ELISA-based test can improve the throughput of this test and lead to a large-scale study to characterize the clinical picture of PLF patients and management of this medical conditions.

## Abbreviations

PLF: (perilymphatic fistula)
CTP: (Cochlin-tomoprotein)
hCTP: (human CTP)
